# Urbanization enhances body condition, but not innate immune defences, in a common waterbird

**DOI:** 10.1098/rsos.241062

**Published:** 2025-01-15

**Authors:** Amelia Chyb, Kevin D. Matson, Radosław Włodarczyk, Joanna Drzewińska-Chańko, Piotr Minias

**Affiliations:** ^1^ Department of Biodiversity Studies and Bioeducation, University of Lodz, Faculty of Biology and Environmental Protection, Banacha 1/3, Lodz 90-237, Poland; ^2^ Wildlife Ecology and Conservation Group, Wageningen University & Research, Droevendaalsesteeg 3a, Wageningen 6708PB, The Netherlands

**Keywords:** innate immunity, Eurasian coot, birds, urban-rural, health

## Abstract

There is a growing body of evidence that urbanization can affect body condition and immune function in wild birds, although these effects may be complex and taxa-specific. Here, we assessed the effects of urbanization on body condition (size-corrected body mass and haemoglobin concentration) and innate immune defences (haemolysis–haemagglutination assay, haptoglobin concentration and bacterial killing assay) in 136 Eurasian coots (*Fulica atra*) from three urban and three non-urban populations across Poland. We also quantified the heterophil to lymphocyte ratio to control for the potential effect of physiological stress on immune defences. We found that urban coots showed significantly better condition than non-urban ones. At the same time, we found no relationship between any immune defence and urbanization or condition. Thus, our study offers no support for condition-dependent immune function. Our analyses also revealed significant differences between male and female coots in both condition and immune defences; however, we found no evidence for sex-specific responses to urbanization. In conclusion, our study provides correlative evidence that urban habitat enhances condition, but not immune defences in the Eurasian coot.

## 1. Introduction

Rapid human population growth and industrialization are associated with intensive land transformation and progressing urbanization [1]. Urban landscapes create novel habitats and shape animal communities [2]. Many animal species adapt to human-induced changes in landscape structure and benefit from conditions specific to urban environments, such as increased (anthropogenic) food sources, milder microclimate and relaxed predation pressure [3–5]. One of the significant features of urban habitats is altered pathogen pressure. On the one hand, the absence or low abundance of suitable vectors and disruptions in pathogen or vector life cycles can result in reduced pathogen pressure in urban environments [6]. On the other hand, high densities of urban populations can facilitate direct transmission of pathogens, and landscape transformations promote new interactions among species and the emergence of novel pathogens [7]. Both of these can lead to elevated pathogen pressure in urban areas. Changes in pathogen pressure along urbanization gradients may directly lead to adaptation and acclimatization in the host immune system, either reflecting genetic divergence between urban and non-urban populations or plastic responses (e.g. condition-dependent downregulation or upregulation of immune defences). However, so far, there are limited empirical data on whether and how differences in nutritional or physiological condition of animals in urban and non-urban areas may relate to investment in immune defences.

While differences in body condition between urban and non-urban birds are well documented (e.g. [8,9]), the mechanisms underlying these differences are not. Body condition may be affected by environmental conditions (e.g. food availability or pollution levels) that vary along an urban gradient [10] independently of pathogen or parasite pressure. Greater food availability in urban habitats may reduce starvation risk, elevate overwinter survival rate, increase post-winter body condition and positively affect bird reproductive performance [2]. However, benefits from access to predictable (but often low-quality) anthropogenic food resources may be counterbalanced by strong intraspecific competition and high population densities in urban birds [11,12]. Thus, bottom-up ecological processes in urban areas may increase the abundance of poor-quality individuals, which are unable to effectively maintain body resources crucial for successful breeding and survival in the periods of unfavourable environmental conditions, such as long periods of low temperatures or precipitation [2,11].

Although body condition in wild-living animals is traditionally quantified as the size of energy reserves [13], there is a broad spectrum of haematological and biochemical indices that measure different physiological components contributing to an overall condition. One such index is total blood haemoglobin concentration, a well-studied haematological parameter that reflects blood oxygen capacity and aerobic performance of an organism [14]. Total haemoglobin concentration has been commonly used as an indicator of physiological condition in a wide range of eco-immunological studies on birds, mostly passerines [15]. However, it is also acknowledged to provide reliable measurements of condition across diverse non-passerine bird orders [16]. This index correlates with diet quality, survival and several reproductive parameters, supporting its robustness as an indicator of physiological condition in both wild and captive birds (reviewed in [14]). A wide range of studies have investigated relationships between urbanization and condition parameters in diverse groups of birds; however, the results are highly inconsistent, showing either positive [17–19] or negative associations [8,20,21]. Urbanization may also have contrasting effects on different components of condition [22,23]. For example, urban noisy miners, *Manorina melanocephala,* had higher body mass but lower whole blood haemoglobin concentration in comparison to non-urban conspecifics [24]. Taking all this into account, processes governing condition in urban bird populations may be considered highly complex and multi-faceted.

Urban environments can shape the immune system of inhabitants via several potentially interacting mechanisms. For example, urbanization-related variation in physiological condition can indirectly modulate the levels of immune defences. On the one hand, higher body condition (e.g. maintained by easy access to rich anthropogenic food sources) can enable greater investments in the immune system. On the other hand, an urban environment may not be sufficient to allow effective upregulation of the immune function, as poor-quality anthropogenic food sources may be deficient in key nutrients, despite their high calorific value [25]. Also, the strength of the immune defences in urban birds can be limited by many factors, such as stress or pollution [26,27]. Finally, immune system maintenance and use may be resource expensive, and suppression of immune function in urban habitats with low pathogen and parasite pressure may be adaptive, allowing individuals to shift limited resources to other activities, such as reproduction [28]. These mechanisms may be particularly impactful on the innate immune system, which is generally unaffected by the previous exposure to pathogenic agents [29]. Natural antibodies (NAbs; mostly pentameric immunoglobulin M) and the complement system are important first lines of defence against parasites [30]. Some urban birds exhibit reduced innate immune defences (e.g. lower levels of natural antibody-mediated agglutination, complement-mediated lysis, acute phase proteins such as haptoglobin and bacterial killing capacity; e.g. [27,31] but see [32,33]), but whether these reductions reflect adaptations to reduced pathogen pressure or constraints of an urban environment (e.g. stress) is uncertain.

In general, urban stressors may not only suppress immune function, but they may also promote alterations in certain immunological components and, in fact, some indicators of physiological stress may have mechanistic links to the immune system [34,35]. For example, the proportion of two leukocyte types, that is heterophils to lymphocytes (H/L ratio), is often used as a simple measure of physiological stress in birds [36]. The level of heterophils in peripheral blood increases with stress to provide defences against infection through possible injury, while the number of lymphocytes in peripheral blood decreases, as they are either mobilized into tissue surveillance pathways or moved to source organs (e.g. bone marrow and spleen) and the marginated pool [37]. These changes occur through the process of white blood cell trafficking between bone marrow, blood and internal organs, which is initiated by the release of catecholamine and glucocorticoid stress hormones [37]. Many studies of passerine bird species point to an elevated physiological stress in birds from urban populations (e.g. [24,38,39]); it remains to be tested if a similar pattern prevails in other (non-passerine) avian orders.

**Figure 1.**
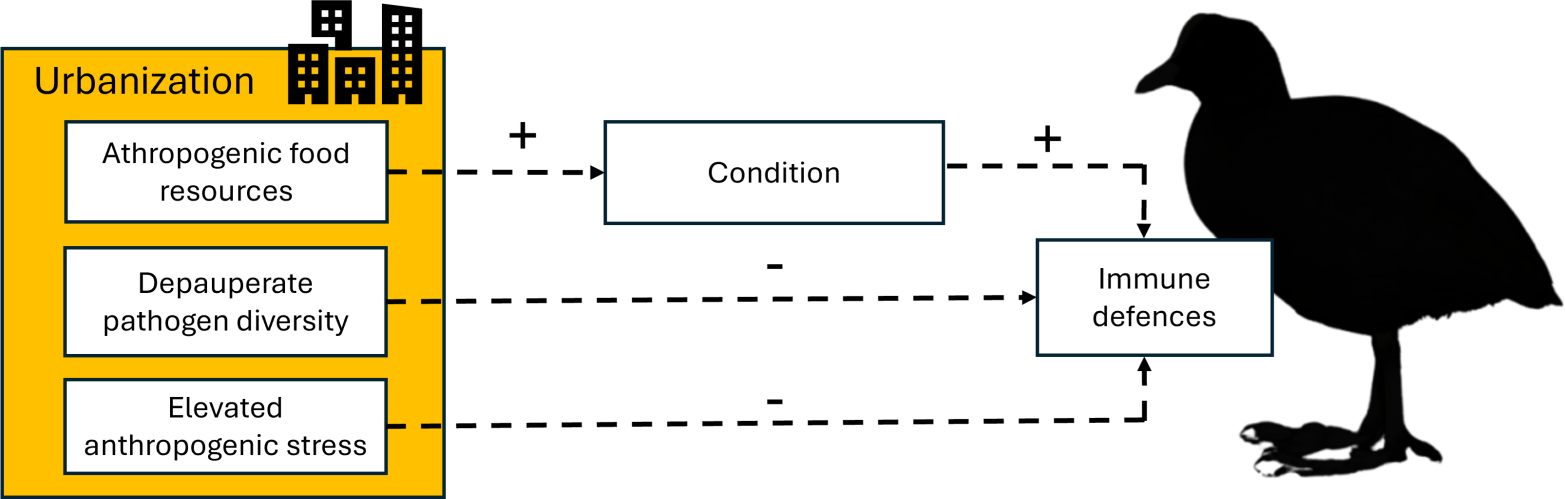
Conceptual framework showing predictions on the associations between urbanization, condition and immune defences in the Eurasian coot.

The aim of this study was to examine associations between urbanization and condition, stress and innate immune function in a common non-passerine waterbird, the Eurasian coot, *Fulica atra*. For this purpose, we sampled 136 breeding coots from three urban and three non-urban populations in central Europe (Poland). Body condition was assessed with both morphological (body mass controlled for structural size) and haematological (total blood haemoglobin concentration) parameters, physiological stress was assessed with H/L ratio and innate immune function was assessed with three indices (haemolysis–haemagglutination assay, bacterial killing assay (BKA) and haptoglobin). We aimed to test several alternative hypotheses. First, we hypothesized that urban coots would have higher condition compared with non-urban ones (possibly owing to anthropogenic food sources and milder microclimate; figure 1). Second, we hypothesized that higher condition in urban areas could promote higher levels of innate immune defences, assuming that greater defences in urban habitats are adaptive (figure 1). Alternatively, greater innate immune defences may not be required under depauperate parasite communities in urban areas, resulting in reduced or similar innate immune defences (despite possibly elevated condition). Another alternative is that any investment in innate immune defences by urban birds may be effectively constrained or masked by other factors, e.g. elevated stress (figure 1). Under this scenario, we expected to observe higher stress (H/L ratio) and condition, but not necessarily immune defences in urban than non-urban coots. Finally, we hypothesized that urbanization can modulate stress, condition and immune defences in a sex-specific manner, reflecting different resource allocation strategies and different regimes of sexual selection between males and females [40]. Sexual dimorphism in immunological parameters is a common phenomenon in wild bird populations, as a result of different responses of males and females to environmental conditions [40], possibly including responses to urbanization.

## 2. Material and methods

### 2.1. Study area and general field procedures

**Figure 2.**
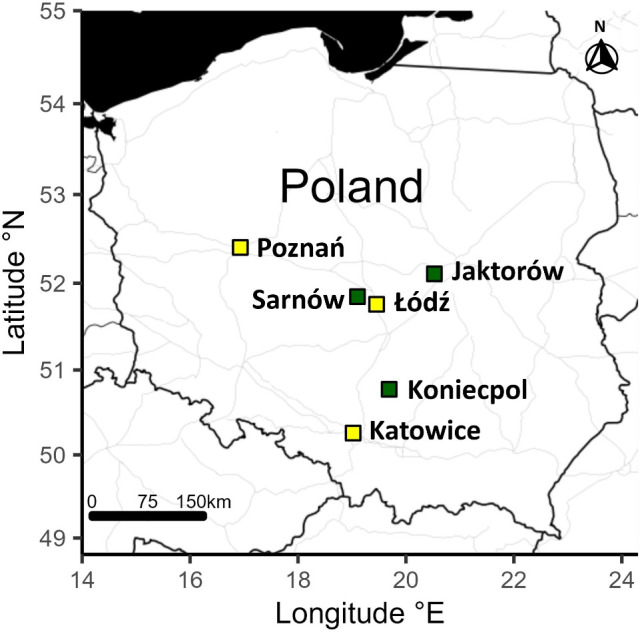
Location of the sampling sites. Urban and non-urban coot populations were marked in yellow and green, respectively.

The study was performed during two breeding seasons (March–July 2021 and 2022) in six populations (three urban and three non-urban) in Poland. Specifically, Eurasian coots were sampled in three large urban agglomerations of Poznań (52°24′30″ N, 16°56′01″ E), Katowice (50°15′30″ N, 19°01′39″ E) and Łódź (51°46′37″ N, 19°27′17″ E). Urban sampling sites included artificial water bodies located in city centres (either in urban parks or densely urbanized residential areas) and characterized by elevated anthropogenic pressure and limited availability of natural reed vegetation. Coots from non-urban populations were captured at three fish pond complexes located in Sarnów (51°50′33″ N, 19°08′36″ E), Koniecpol (50°46′27″ N, 19°41′20″ E) and Jaktorów (52°05′25″ N, 20°32′50″ E) (figure 2). Non-urban sampling sites were characterized by natural-like habitat structure with low human disturbance and extensive areas of reed vegetation (>50% water surface covered with vegetation at each site). All non-urban sampling sites were surrounded mainly by natural or semi-natural areas (e.g. wetlands, agricultural areas, woodland, shrubs and wasteland); man-made structures (e.g. buildings and roads) covered less than 5% of the area within 2 km buffers around these sites (Quantum Geographic Information System; QGIS 3.32.2, QGIS Development Team 2023, Open Source Geospatial Foundation, Beaverton, USA). The mean distance between urban sites was 140 km (ranging from 100 to 182 km), and the mean distance between non-urban sites was 211 km (ranging from 168 to 278 km).

We captured 15−32 adult individuals per population (total sample size: *n* = 136; electronic supplementary material, table S1). Birds were caught with the loop trap made of multifilament fishing line placed on the nest during incubation. Each bird was individually marked with a metal ring (left tarsus) and a plastic neck collar with an alphanumerical code to avoid recaptures. Biometric measurements, including body mass and wing length, were collected from each individual. Using heparinized microcapillary tubes, we collected *ca* 250 µl of blood from the tarsal vein of each individual to obtain plasma for immunological assays. Collected blood was stored at 4°C for up to 8 h and centrifuged (10 000 rpm, 5 min) to separate plasma from the whole blood. Aliquots of whole blood were used for haemoglobin concentration quantification (to assess physiological condition), blood smear preparation (to assess leukocyte profiles) and molecular sexing (whole blood mixed with 96% ethanol). For the latter, genomic DNA was extracted using a DNA Purification Kit (Euryx, Gdańsk, Poland) according to the manufacturer’s protocol and chromo-helicase-DNA-binding genes were amplified following the protocol developed by Griffiths *et al*. [41]. Polymerase chain reaction products were visually examined on agarose gels after horizontal electrophoresis; one or two visible bands indicated a male or a female, respectively.

### 2.2. Condition indices

We used two different condition indices: body mass corrected for structural size (*n* = 136) and total blood haemoglobin concentration (*n* = 134, two measurements failed for technical reasons). Body mass was measured with an electronic scale (± 1 g) in all captured coots. Since both energy reserves (condition) and structural body size contribute to total body mass, in the analyses we controlled for wing length, which was measured with a stopped ruler (± 1 mm). Specifically, wing length was included as a linear predictor of body mass in our statistical models (see §2.8 for details). Total blood haemoglobin concentration was measured with HemoCue Hb 201+ portable photometer (HemoCue, Ängeholm, Sweden) within 1 min from venipuncture using disposable HemoCue cuvettes. The HemoCue Hb 201+ photometer uses a modified azide methaemoglobin reaction and gives robust measurements of total blood haemoglobin concentration in wild birds [42].

### 2.3. Physiological stress

We used leukocyte profiles to assess the physiological responses of coots to urbanization-related stressors. The blood smears were stained using the May–Grünewald–Giemsa method and scanned at 1000× magnification with oil immersion using a light microscope. In each smear, 100 blood cells were randomly chosen and assigned to one of the cell types: heterophils (H), lymphocytes (L), basophils (B), eosinophils (E) and monocytes (M). The H/L ratio was calculated as a proportion of heterophils to lymphocytes and log-transformed owing to strong right skewness (1.52). In total, H/L ratio was quantified for 125 individuals.

### 2.4. Immunological assays

We performed three assays to measure innate immune defences of urban and non-urban coots.

### 2.5. Haemolysis–haemagglutination assay

Haemolysis measures a cell lysis response dependent on the interaction between NAbs and complement, while haemagglutination reflects NAb activity only [43,44]. Haemolysis–haemagglutination assay was performed according to the protocol developed by Matson *et al*. [43]. First, for each individual, 25 µl of plasma was pipetted into the first and second well in each row of the 96-well round-bottom microtiter plate. We added two control samples of chicken plasma to each plate. Next, we serially diluted (1 : 2) the plasma samples using phosphate-buffered saline (PBS; D5652; Dulbecco’s PBS, Sigma Aldrich, USA). The first well was maintained with pure plasma, the second half-strength and so forth, down to the eleventh well. The last (twelfth) well contained pure PBS as a negative control. Next, 25 µl of a 1% suspension of rabbit red blood cells was added to each well using a multichannel pipette. After gentle swirling (30 s) and securing with parafilm, the plates were incubated in the water bath at 37°C for 90 min. Next, the plates were laid at a 45-degree angle and cooled at room temperature for 20 min. Immediately after cooling, the first scan was made using a flatbed scanner. The second scan was made after storing the plates flat for 70 min at room temperature. The first scan was used to score lysis; the second to score haemagglutination. Scanned full-plate images were divided into separate rows, randomized and scored twice. To avoid the reaction of plasma and rabbit blood cells with the surface of the plates, all plates were blocked with a 2% solution of powdered milk in PBS, washed with the solution of Tween in PBS, air-dried and stored in the freezer (−18°C) before conducting the assay.

### 2.6. Haptoglobin concentration

Haptoglobin is an acute-phase protein that binds free haemoglobin released from damaged red blood cells, preventing iron loss and kidney damage [45]. We used a commercially available colourimetric assay to quantitatively measure the concentration of haptoglobin in collected plasma samples (TP-801; PHASE Haptoglobin Assay, Tridelta, Maynooth, Ireland). Following the manual protocol, we transferred 7.5 µl of each collected coot plasma sample per well. Per 96-well flat-bottom microtiter plate, we also included 7.5 µl of standard chicken plasma, a range of diluted haptoglobin standard (from 0.312 to 2.5 mg ml^−1^), and a blank sample (0.0 mg ml^−1^). Haptoglobin standards, a blank sample and a pooled chicken plasma sample were placed on each plate twice (in the first and the last column), following the protocol of the manufacturer. After adding the first reagent (stabilized haemoglobin), we made pre-scans to measure the absorbance at two wavelengths (450 and 630 nm), which allowed to correct for the differences in redness, colour and cloudiness of the plasma samples in the downstream analyses. The final scan (at 630 nm) was made after 5 min of sample incubation at 30°C with chromogen reagent, which initiates the colour-changing reaction. The measurements of the haptoglobin standard dilution series were used to generate the calibration curve, allowing calculations of haptoglobin concentration in all other wells. The haptoglobin assay was carried out for 135 individuals (one sample was excluded because of insufficient plasma volume) spread randomly over two plates.

### 2.7. Bacterial killing assay

The BKA is a simple functional *in vitro* assay of the capacity of plasma samples to kill (or limit the growth of) a standard microorganism challenge [46]. The initial working solution (IWS) of *Escherichia coli* (ATCC no. 8739) was prepared with stock solutions made from commercially available lyophilized pellets (3.1 × 10^7^ colony-forming units (CFUs) per pellet; Epower Microorganisms no. 0483E7, MicroBioLogics, St Cloud, MN) according to the instructions provided by the manufacturer. Next, we diluted 20 µl of IWS in 200 µl of sterile PBS. To estimate the concentration of CFUs in the diluted IWS, we spread 75 µl of the mixture onto duplicate sterile tryptic soy agar plates and incubated them at 37°C for 8−16 h (i.e. until individual colonies were well sized for accurate counting). After incubation, we counted the number of bacterial colonies on each plate and adjusted IWS until the final concentration was *ca* 200 CFUs per75 µl. Fifty-microlitre aliquots of each coot plasma sample were diluted in 150 µl of sterile PBS and incubated with 20 µl of IWS at 41°C. Next, in duplicate, 75 µl of the incubated mixtures were spread onto sterile agar plates, which were left to dry and then covered and stored upside down in the incubator for 8−16 h at 37°C. For each batch of tested samples, negative and positive controls were prepared in duplicate as either 200 µl of pure PBS (to exclude contamination) or 20 µl of IWS diluted in 200 µl of PBS, respectively. After incubation, the plasma killing capacity was calculated by dividing the mean number of bacterial colonies on duplicated sample plates by the mean number of bacterial colonies on the corresponding duplicated positive control plates. The BKA was carried out for 129 individuals (seven samples were excluded owing to insufficient plasma volume).

### 2.8. Statistical analysis

First, we used general linear models (GLMs) to test for differences in condition indices, physiological stress and innate immune parameters between urban and non-urban populations of the Eurasian coot. All the measures of condition (body mass and blood haemoglobin concentration), physiological stress (log H/L ratio) and innate immunity (haemagglutination, haemolysis, haptoglobin concentration and bacterial killing capacity) were entered as response variables in separate models. Urbanization (urban versus non-urban), population identity nested in urbanization level (three urban and three non-urban populations were sampled) and sex were entered as fixed factors, while wing length was entered as a covariate. We also included an interaction between urbanization and sex to test whether males and females responded differently to urbanization in terms of condition, physiological stress and immune responses. Second, we ran GLMs to test for associations of innate immune parameters with condition and physiological stress. Here, haemagglutination, haemolysis, haptoglobin concentration and bacterial killing capacity were used as response variables, while body mass, haemoglobin concentration and log H/L ratio were entered as covariates. As in the previous models, sex was entered as a fixed factor, and wing length was entered as an additional covariate. We also included interactions between sex and condition parameters to test whether males and females differed in their relationships between condition and immune defences. In all models, year was included as a fixed factor. Highly non-significant (*p* > 0.15) predictors were removed (in one step) to obtain more parsimonious reduced models. Full and reduced model results are presented. The effect sizes were calculated as partial eta-squared (*η*^2^_p_) [47]. All computations were performed in the *glmmTMB* package [48] developed for R v. 4.0.3 statistical environment (R Foundation for Statistical Computing, Vienna, Austria) and Statistica v. 13.1 software (TIBCO Software Inc., Palo Alto, CA).

## 3. Results

**Figure 3.**
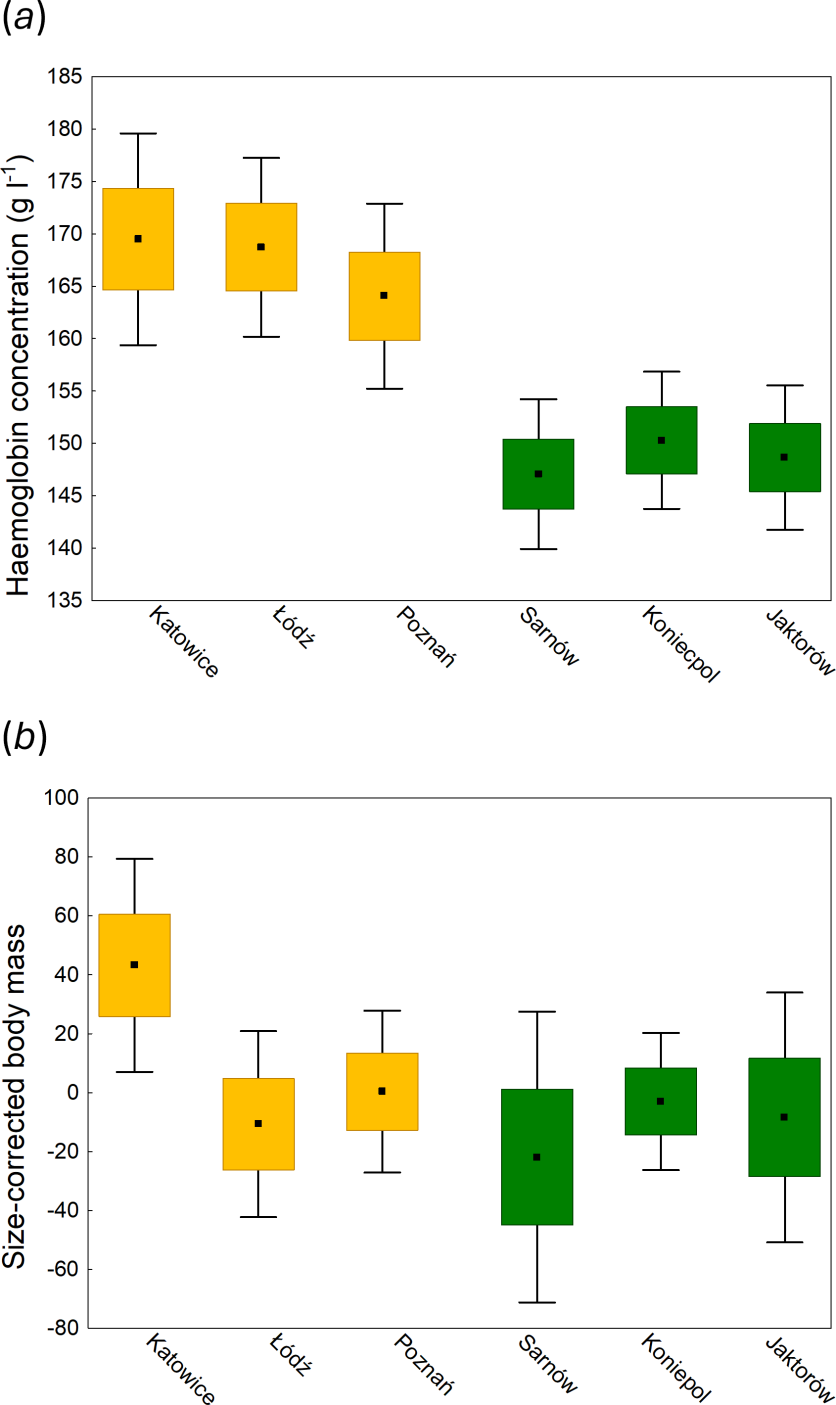
Differences in body condition in three urban (yellow) and three non-urban (green) populations of the Eurasian coot. Means (central point), s.e. (box) and 95% confidence intervals (whiskers) of haemoglobin concentration (*a*) and size-corrected body mass (*b*) are shown. For the purpose of visualization, size-corrected body mass was calculated as residuals from body mass against wing length.

Urbanization was identified as a significant predictor of body condition in the Eurasian coot. After controlling for variation in wing length (structural body size), urban birds showed higher haemoglobin concentration (*β* = 7.27 ± 1.56, *p* < 0.001; table 1; figure 3*a*) and body mass (*β* = 31.04 ± 20.71, *p* = 0.010; table 2; figure 3*b*) than non-urban birds. Urbanization explained 14.3 and 5.1% of variation in these parameters, respectively. The effect of population (nested in urbanization level) was non-significant in the analysis of haemoglobin concentration (*p* = 0.82; table 1), suggesting a consistent effect of urbanization on this component of condition. By contrast, population was significant in the case of body mass (*p* = 0.044; table 2), suggesting greater variation between specific locations within each urbanization level. At the same time, we found no differences in H/L ratio between urban and non-urban birds (*p* = 0.86; electronic supplementary material, table S2), providing no evidence for the effect of urbanization on physiological stress in coots.

**Table 1.** Models assessing the relationship of blood haemoglobin concentration with urbanization level across six populations of the Eurasian coot. (The population effect was nested in the level of urbanization. Significant predictors are marked in bold.)

predictor	coefficient (mean ± s.e.)	*F*	*p*
*full model*			
**intercept**	**124.39** ± **57.25**	**4.70**	**0.032**
**urbanization level**	**12.55 ± 5.72**	**17.45**	**<0.001**
**sex (M**)	**14.81 ± 4.35**	**11.60**	**<0.001**
**year**	**−8.96 ± 3.47**	**6.66**	**0.011**
wing length	0.17 ± 0.26	0.42	0.52
population	n.a.	0.38	0.82
*reduced model*			
**intercept**	**157.76 ± 1.46**	**11 639.82**	**<0.001**
**urbanization level**	**7.27 ± 1.56**	**21.75**	**<0.001**
**sex (M**)	**8.32 ± 1.45**	**32.71**	**<0.001**
**year**	**−4.48 ± 1.56**	**8.23**	**0.005**

**Table 2.** Models assessing the relationship of body mass with urbanization level across six populations of the Eurasian coot. (The population effect was nested in the level of urbanization. Significant predictors are marked in bold.)

predictor	coefficients (mean ± s.e.)	*F*	*p*
*full model*			
intercept	−125.34 ± 213.83	0.57	0.45
**urbanization level**	**30.86 ± 20.79**	**5.15**	**0.025**
**sex (M**)	**128.58 ± 16.18**	**63.16**	**<0.001**
**wing length**	**4.37 ± 0.98**	**20.03**	**<0.001**
population	n.a.	2.44	0.051
year	−3.55 ± 12.82	0.08	0.78
*reduced model*			
**intercept**	**−132.85 ± 211.33**	**0.62**	**0.43**
**urbanization level**	**31.04 ± 20.71**	**6.86**	**0.010**
**sex (M**)	**128.18 ± 16.05**	**63.75**	**<0.001**
**wing length**	**4.40 ± 0.97**	**20.62**	**<0.001**
**population**	**n.a.**	**2.52**	**0.044**

Urban and non-urban birds showed similar innate immune defences. We found no immunological differences in relation to urbanization (all *p* > 0.05; electronic supplementary material, table S3), although two of the four immunological parameters did show associations with either physiological stress or condition (electronic supplementary material, table S4). Haptoglobin concentration was positively associated with physiological stress (H/L ratio; *β* = 0.68 ± 0.23, *p* = 0.003) but showed no relationship with body mass (*p* = 0.27) or haemoglobin concentration (*p* = 0.16; electronic supplementary material, table S4). Haemolysis was negatively associated with body mass (*β* = −0.002 ± 0.001, *p* = 0.040) but showed no relationship with haemoglobin (*p* = 0.20) or physiological stress (H/L ratio; *p* = 0.09; electronic supplementary material, table S4). No interaction between sex and any condition parameter was significant (*p* > 0.05), and all were removed.

**Figure 4.**
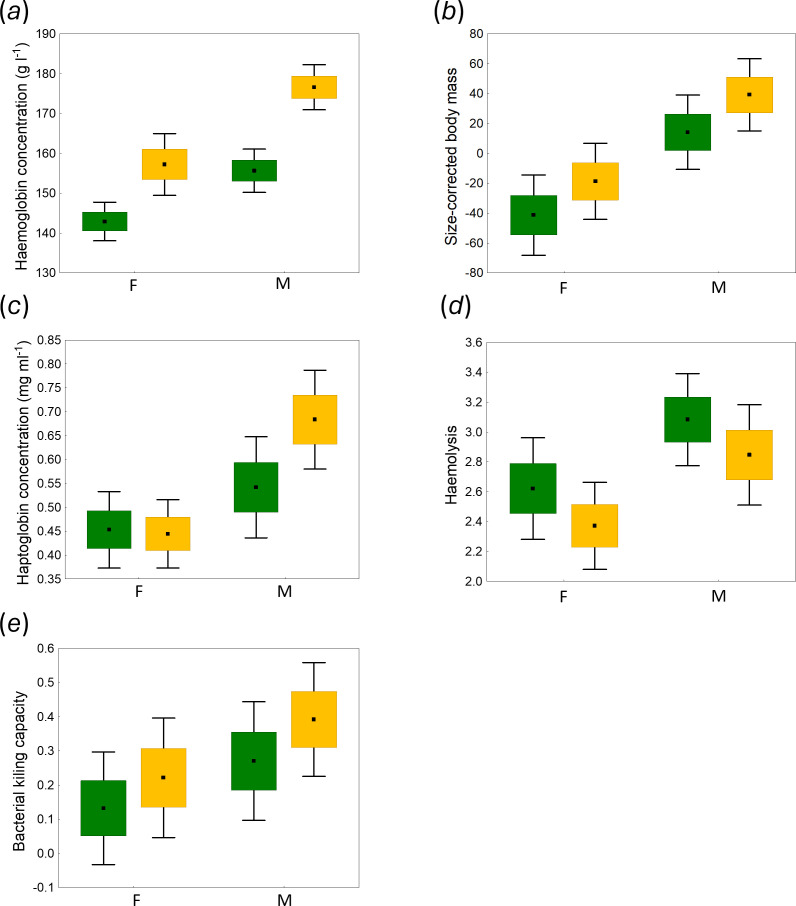
Differences in body condition and immune defences between males (M) and females (F) in urban (yellow) and non-urban (green) populations of the Eurasian coot. Means (central point), s.e. (box) and 95% confidence intervals (whiskers) of haemoglobin concentration (*a*), size-corrected body mass (*b*), haptoglobin concentration (*c*), haemolysis (*d*) and bacterial killing capacity (*e*) are shown.

Finally, we found significant differences between sexes in both condition and immunological parameters (table 3; figure 4). Male coots had significantly higher body condition, both in terms of blood haemoglobin concentration and body mass controlled for wing length (both *p* < 0.001; tables 1 and 2). Similarly, males had higher levels of three of the four immune indices: haemolysis (*β* = 0.84 ± 0.26, *p* = 0.002), haptoglobin (*β* = 0.16 ± 0.05, *p* = 0.001) and bacterial killing capacity (*β* = 0.18 ± 0.07, *p* = 0.016; electronic supplementary material, table S4). However, we found no evidence for the effect of urbanization on shaping the differences between sexes (neither in condition nor in immunological defences): none of the interactions between urbanization and sex were significant (*p* > 0.05), and all were removed from the full models.

**Table 3.** Summary statistics for condition indices, heterophil/lymphocyte (H/L) ratio and immunological parameters in male and female Eurasian coots.

trait	females				males			
	mean	s.e.	range	*n*	mean	s.e.	range	*n*
blood haemoglobin concentration (g l^−1^)	150.17	2.41	117–210	65	167.48	2.32	129–218	69
body mass (g)	686.97	8.35	525–885	66	866.44	9.19	650–1070	70
H/L ratio	0.81	0.065	0.18–2.65	58	0.83	0.056	0.15–2.13	67
haemagglutination	8.67	0.18	5–1	66	8.80	0.15	6.25–11	69
haemolysis	2.50	0.11	0–5.5	66	2.95	0.11	1.5–7.75	69
haptoglobin concentration (mg ml^−1^)	0.45	0.026	−0.059 to 1.41	66	0.62	0.037	0.025–2.05	69
bacterial killing capacity	0.17	0.059	−0.87 to 1.0	63	0.34	0.06	−0.40 to 1.0	66

## 4. Discussion

Our study provides evidence for a positive association between urbanization and condition in the Eurasian coot, as we found that individuals from urban populations have significantly higher haemoglobin concentration and size-corrected body mass than non-urban ones. At the same time, we found no differences between coots from urban and non-urban populations in terms of either physiological stress (H/L ratios) or innate immune defences. These results lend support to the hypothesis that urban coots, despite elevated condition, maintain similar investment in innate immune defences as non-urban ones. Finally, our results showed significant differences between sexes in both condition indices and most of the measured immune defences; however, we found no evidence for sex-specific responses of these parameters to urbanization.

Specific environmental conditions of human-dominated areas may affect various phenotypic characteristics of wild-living animals, including body size, body condition and health [5]. Variation in bird condition along urban gradients could be shaped simultaneously by a wide range of external and internal factors [8,9]. Many studies show that urban individuals can be in poorer [9,49–51], similar [52] or better [8,17,34] condition than non-urban ones, which suggests that observed differences may be species-specific or arise from local environmental conditions. At the population level, poorer average condition in urban birds may be caused by the survival of low-quality individuals, which can satisfy their basic caloric demand with anthropogenic food ([49], but see [8]). On the contrary, studies showing better condition in urban individuals suggest that use of human-subsidized feeding resources may contribute to better nutritional state of the body and favour effective accumulation of energy reserves [34]. In our study, coots from urban populations were consistently characterized with better body condition, as measured with two different parameters, i.e. size-corrected body mass and blood haemoglobin concentration. These results may reflect not only better food availability in human-dominated areas, but also specific behavioural responses of urban coots. In fact, we have shown previously that urban coots were significantly bolder and more willingly exploited anthropogenic food sources in comparison to non-urban individuals [53]. Importantly, differences between urban and non-urban coots were not only apparent in size-corrected body mass, which is a simple proxy of organismal energy reserves, but also in terms of physiology: urban coots had significantly higher blood haemoglobin concentration than non-urban ones. In birds, total blood haemoglobin concentration is primarily correlated with the size and number of erythrocytes and reflects the aerobic capacity of the organism [14]. Higher haemoglobin concentration in urban coots might be related to higher availability of food (preventing birds from starvation), lower risk of blood parasitism or both [34]. Our results also hint at variation in condition among the studied coot populations within particular (urban or non-urban) habitats, as the population effect on body mass was significant in the reduced model. Any such differences between coot populations could be caused by variation in local environmental conditions between specific urban sites, for instance in the trophic conditions of urban water reservoirs or in the availability of food resources provided by humans [54]. Nevertheless, variation between populations was not observed at the physiological level (haemoglobin concentration), which suggests that different condition indices can respond differently to urbanization.

The level of an individual investment in immune defences against pathogens and parasites may vary depending on both the current capabilities and needs of an organism [55]. Many studies have reported associations between nutritional body condition and efficiency of the immune system in birds (e.g. [56–58]). For instance, experimentally induced reduction in body mass was associated with lower T-cell-mediated immune response in yellow-legged gulls *Larus cachinnans* [56]. In our study, despite elevated condition in urban coots, we found no differences between urban and non-urban individuals in innate immune parameters. Hence, it seems likely that despite available internal resources, urban coots may incur little benefits from elevated investment in innate immune defences. Relaxed pathogen pressure in human-dominated environments might also reduce the need for sustained upregulation of the immune system [6] and, in fact, there is accumulating evidence for depauperate diversity of urban pathogen communities [31,34,59–61]. In our study, coots were sampled during the breeding season while bearing reproductive costs [62]. Without the need for additional investments in immune defences, improved body condition of urban birds might signal a redirection of urban-derived resources (i.e. energy and biochemical substrates) to other activities (e.g. reproduction), ultimately increasing fitness in urban-dwelling individuals [28]. These results are well aligned with our previous work on the major histocompatibility complex (MHC) genes in an urban population of the Eurasian coot [63]. The MHC forms the main pathogen-recognition component of acquired immunity, and individual MHC diversity determines the spectrum of non-self (pathogenic) antigens recognized by the acquired immune system [64]. Urban coots showed a negative relationship between MHC diversity and several fitness-related traits, suggesting that the costs of maintaining a broad MHC repertoire in urban habitats may outweigh benefits [63]. Taking all this into account, we cannot exclude that upregulation of both innate and acquired immune defences may not be adaptive in urban coots.

Via elevated levels of glucocorticoid steroids, chronic stress may suppress immune function and increase susceptibility to infection [65]. Therefore, any positive association between body condition and immunological defences may be constrained or masked by physiological stress in urban-dwelling birds. Indeed, some studies have shown elevated stress levels in urban individuals, as measured with H/L ratios [34] or corticosterone concentrations [66,67]. This elevation probably reflects a response to multiple stressors generated by the human-dominated environment (e.g. elevated human disturbance, noise and light pollution). However, such elevations are not universal, with some studies reporting no differences in stress measures between the urban and non-urban landscape [23,34,50]. Thus, physiological responses to urbanization apparently differ among species. Here, we found no evidence for differences in physiological stress (H/L ratios) in coots from urban and non-urban habitats. Therefore, the absence of an urbanization effect on immune defences in coots was probably not the result of any stress-related constraints.

Females and males may react differently to environmental stressors owing to sex-related differences in body size, physiology or reproductive investment [68,69]. In our study, both sexes showed similar responses (or lack thereof) to urbanization in terms of body condition and immune defences. Despite this, males overall showed higher levels of immune defences than females. Thus, our results suggest that at least some immune defences might be downregulated in females during the breeding season, a possible consequence of greater investment in reproductive processes and resource allocation trade-offs in females ([28,70], but see [40]). This scenario is also supported by the better body condition of male coots compared with females. However, sex-related differences in condition may also be promoted by competitive hierarchies that are dominated by structurally bigger (by 5–10%) males [71]. Larger and often more aggressive males might effectively outcompete females and monopolize food resources within territories. Male coots are also expected to be more effective at competing (via direct interference) with other waterbirds (e.g. ducks) compared with female coots.

In conclusion, our study provides convincing correlative evidence that urbanization promotes better physical and physiological condition in the Eurasian coot. However, we found no support for the hypothesis that elevated condition translates to more robust innate immune defences in urban-dwelling individuals. Considering the similar levels of physiological stress in urban and non-urban coots, we conclude that the absence of urbanization-related differences in immune defences is not the result of stress-related constraints. While our study provides new insights into whether and how urbanization affects condition and immune status in wild birds, we also emphasize the need to further investigate the complex associations between urbanization processes and key aspects of avian immunology and physiology, particularly in poorly studied non-passerine species.

## Data Availability

The datasets supporting this article are available through Dryad [72]. Supplementary material is available online [73].
